# Accelerated aging in people experiencing homelessness: A rapid review of frailty prevalence and determinants

**DOI:** 10.3389/fpubh.2023.1086215

**Published:** 2023-03-16

**Authors:** Rhys Mantell, Ye In Jane Hwang, Kylie Radford, Silvija Perkovic, Patricia Cullen, Adrienne Withall

**Affiliations:** ^1^School of Population Health, Faculty of Medicine and Health, University of New South Wales (UNSW), Sydney, NSW, Australia; ^2^UNSW Ageing Futures Institute, University of New South Wales (UNSW), Sydney, NSW, Australia; ^3^School of Psychology, Faculty of Science, University of New South Wales (UNSW), Sydney, NSW, Australia; ^4^Neuroscience Research Australia (NeuRA), Sydney, NSW, Australia; ^5^The George Institute for Global Health, University of New South Wales (UNSW), Sydney, NSW, Australia; ^6^Ngarruwan Ngadju: First Peoples Health and Wellbeing Research Centre, University of Wollongong, Wollongong, NSW, Australia

**Keywords:** frailty, homelessness, marginalized and vulnerable groups, accelerated aging, cognitive impairment, social determinants of health

## Abstract

**Introduction:**

Older people experiencing homelessness (PEH) are a rapidly growing population at risk of accelerated aging and the early onset of geriatric conditions. One construct that shows promise in predicting age-related decline is frailty. Better understanding the rates and causes of frailty in PEH may improve understanding of its antecedents, thereby facilitating more targeted health and aged care service interventions. The aim of this study was to conduct a rapid review on the prevalence and determinants of frailty in adult PEH.

**Methods:**

We conducted a rapid review of primary research papers studying PEH and frailty or frailty-related concepts.

**Results:**

Fourteen studies were included, which indicate that frailty presents earlier and at higher rates in PEH than community-dwelling cohorts. A notable difficulty for many aging PEH was early-onset cognitive impairment which was associated with a range of negative functional outcomes. Another recurrent theme was the negative impact that drug and alcohol use and dependence can have on the health of PEH. Further, psychosocial and structural determinants such as loneliness, living in an impoverished neighborhood and being female had statistically significant associations with frailty and functional decline in PEH.

**Discussion and implications:**

PEH in their 40s and 50s can be frail and experience geriatric conditions, including cognitive impairment. Factors that have important relationships to frailty and functional decline in PEH include cognitive deficits, drug and alcohol dependence and loneliness, as well as upstream determinants such as gender and ethnicity. More targeted data and research on these factors, including cohort studies to better investigate their potentially causal effects, is important for researchers and practitioners assessing and treating frailty in PEH, particularly those interested in early intervention and prevention.

**Prospero registration ID:**

CRD42022292549.

## Introduction

People experiencing homelessness (PEH) often face challenging living conditions and endure a complex interplay of health and social deprivation. The disadvantage facing PEH has previously been shown by the high rates of early morbidity and mortality that the group faces (1). Studies report mortality rates for PEH 3-to-12 times higher than the age-standardized general population rate (2–4). The burden facing PEH becomes particularly evident as individuals age, where physical and cognitive conditions become more common (1). Approximately two-thirds of older PEH in high-income countries have multiple physical health problems, most commonly cardiac disease, hypertension, diabetes and respiratory illness (5). A recent meta-analysis by Suh et al. (1) found that PEH experience higher rates of geriatric conditions at a younger age compared to community-dwelling adults. Unpacking the various health and social difficulties faced by older PEH is becoming increasingly important (6) as the number of older people in this situation is growing rapidly worldwide.

The cumulative disadvantage experienced by older people who are homeless has led many researchers, clinicians and policy makers to conclude that PEH are at risk of experiencing “accelerated aging,” and consequently the early onset of geriatric conditions such as falls, functional and cognitive impairment, incontinence and immobility (1). There is no standard definition for accelerated aging, but it is generally recognized as a process where a person's physiological system deteriorates earlier and/or more rapidly than when compared to other people or cohorts of comparable age. There is evidence that the pathophysiology that causes this dysregulation is not necessarily related to a specific disease but to a cumulative process of physiological decline, or underlying biological alteration, which is caused by a combination of genetic, environmental and behavioral factors over time (7). Thus, the concept of accelerated aging is often used to examine the cumulative disadvantage of marginalized groups with relatively high morbidity and mortality who seem to “grow old before their time.” In accordance with this, PEH are often considered “older” once they reach the age of 50 (1), as opposed to 65 years which is the nominal existing cut-off for aged care services in many countries.

The implications of accelerated aging can be particularly costly for PEH considering their challenging living environments, the lack of autonomy to modify these environments and the persistent barriers to regular service access that these environments can create or reinforce. In a group that is aging unequally, the concept of early intervention to reduce or slow the onset of geriatric conditions becomes increasingly important. However, one of the main obstacles to early identification and support for accelerated aging in PEH is effectively measuring, unpacking and responding to the underlying, often intersectional, causes of premature geriatric issues in such a diversely disadvantaged cohort (8).

### Frailty as a construct to measure age-related decline

To more effectively identify the early signs of age-related decline, one construct that has gained considerable traction in recent decades is “frailty” (8). Although there is debate about an acceptable definition for the term, frailty can be broadly described as a decreased resilience to stressors, which renders people more vulnerable to disease, disability, hospitalization and social change (6). Similar to the concept of accelerated aging, the pathways that cause frailty are complex and multidimensional. However, unlike accelerated aging, frailty is readily measurable, with a number of validated frailty measures shown to predict various aging outcomes. In a study by Ritt et al. (9), it was found that frailty was a better predictor than disability for overall mortality. Likewise, in Bagshaw et al. (10), those who were frail were more likely to require ongoing help to live at home and also had higher in-hospital mortality compared to non-frail people. In other studies, frailty measures have outperformed chronological age as a predictor of mortality, disability, and cognitive decline, highlighting the relative sensitivity of the construct at capturing “biological” aging (11, 12). For these reasons frailty appears to be a useful approximation of accelerated aging, and may help to detect and/or unpack the complex causes of biological decline, which ultimately lead to the premature onset of geriatric conditions, disability and death (7).

**Figure 1:**
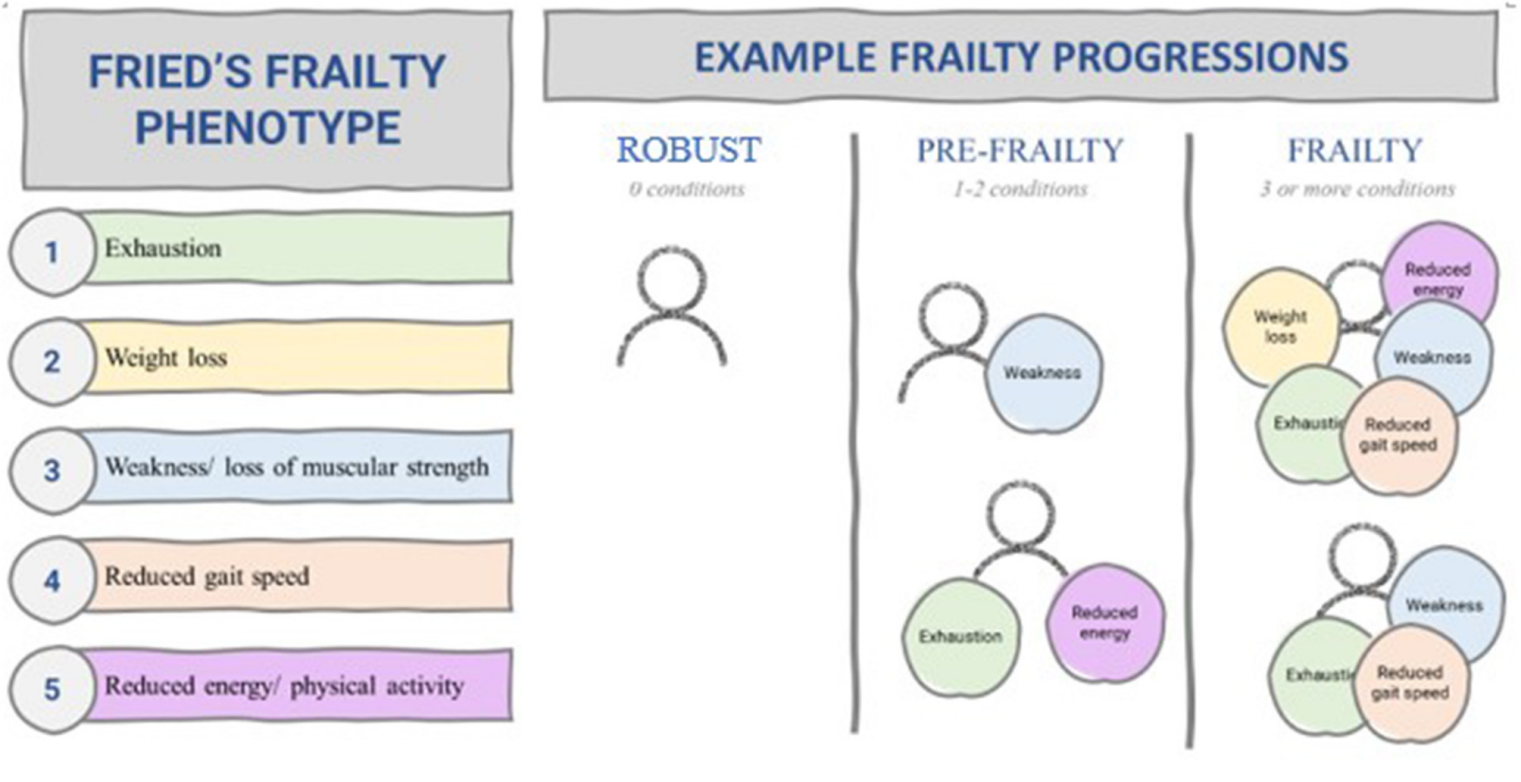
Fried's phenotype model of frailty (13).

Debates about how to measure and operationalize frailty have led to a variety of measures, frameworks and models (8). However, most measures stem from two dominant constructs: the phenotype model and the cumulative deficit model (6, 8). The phenotype model was developed by Fried et al. (13) through clinical observation and epidemiological research and operationalizes frailty as the presence of three or more of the following criteria: exhaustion, weight loss, weakness/loss of muscular strength, reduced gait speed and reduced energy/physical activity (Figure 1).

**Figure 2:**
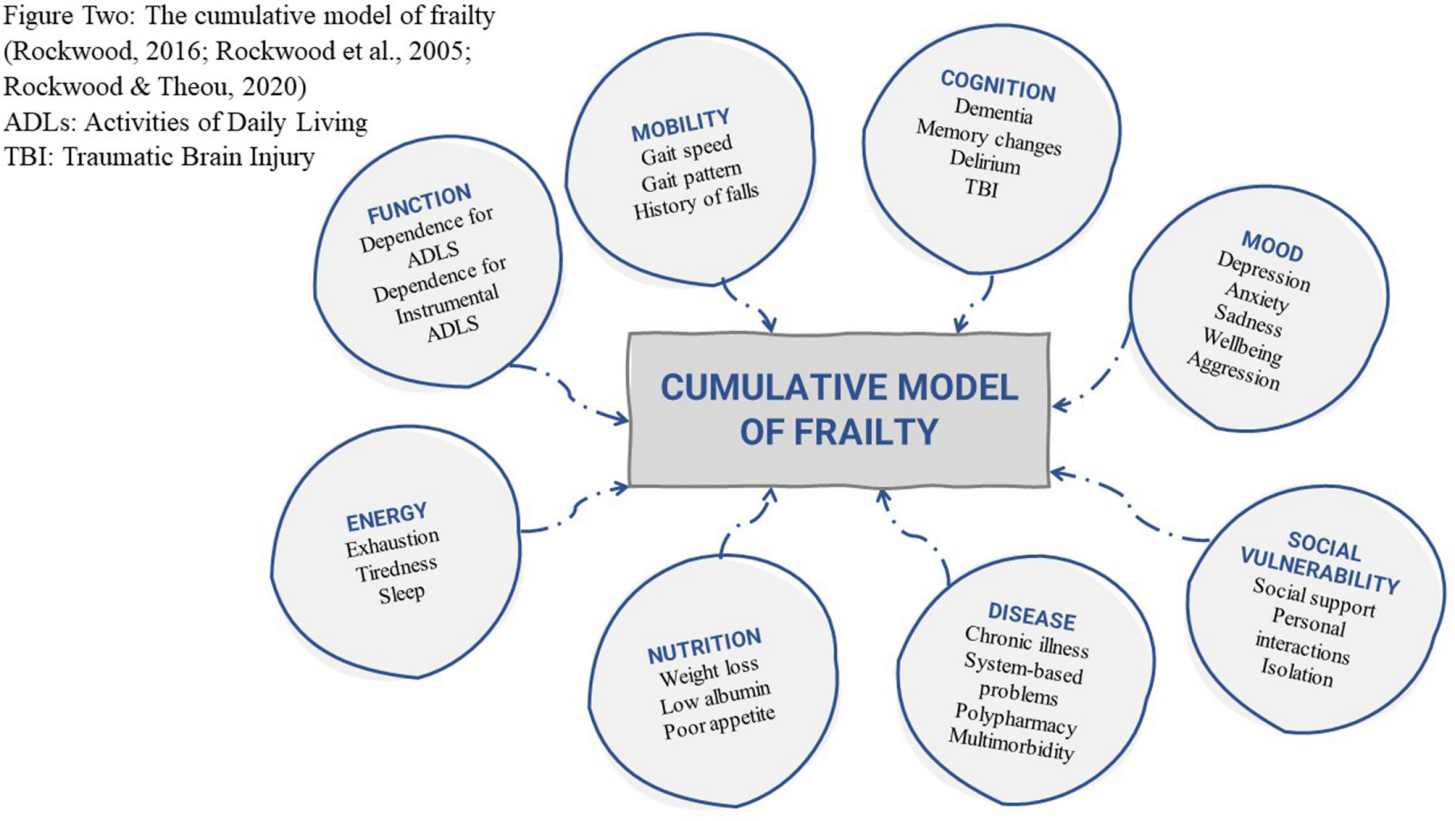
The cumulative model of frailty (14–16). ADLs, activities of daily living; TBI, traumatic brain injury.

**Figure 3:**
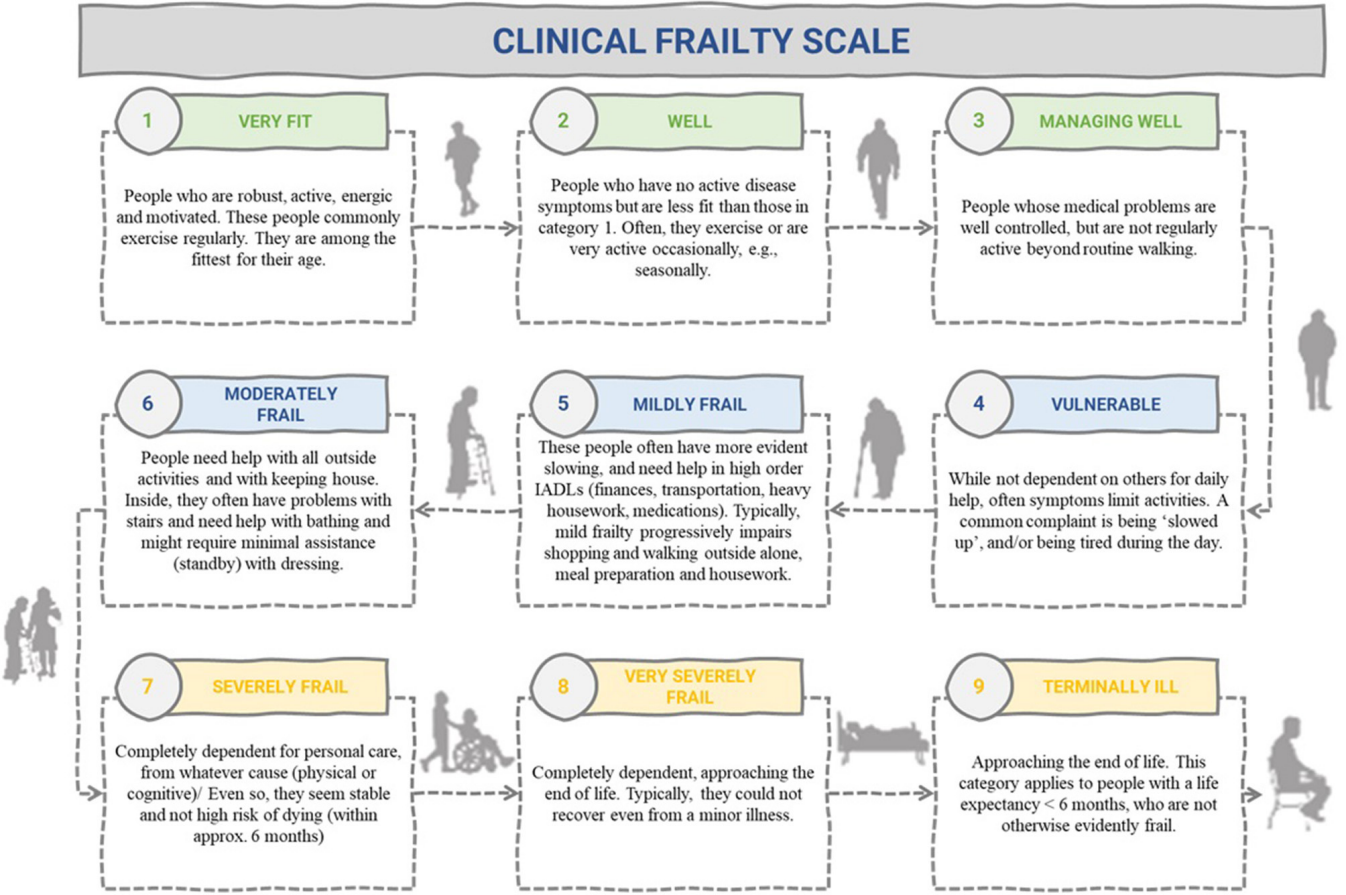
Clinical Frailty Scale (14).

In contrast, the cumulative deficit model was developed by Rockwood et al. (14–16) through consideration of biological theories of aging. It argues frailty to be an accumulation of deficits including clinical signs and symptoms, diseases and disability (Figure 2). This model is often conceptualized as an aggregation of difficulties whereby the more predefined conditions an individual has the more likely they are to be frail (8, 17). In this model, frailty can be measured using a Frailty Index (FI), which for any individual represents the number of concerns present, divided by the number of concerns counted (16). An alternative measure of frailty using the foundations of the cumulative model is the Clinical Frailty Scale (CFS) (Figure 3). Although the CFS uses the concept of cumulative deficits to identify frailty, it is less prescriptive than a Frailty Index approach in determining what is measured and uses clinical judgement to assess a person's baseline health and frailty level (14). The judgment-based CFS is typically advantageous to use when clinicians are available who have experience in the care of older people; whereas the index approach is often useful when experts are unavailable or when a more data-driven measurement approach is desired (14).

**Figure 4:**
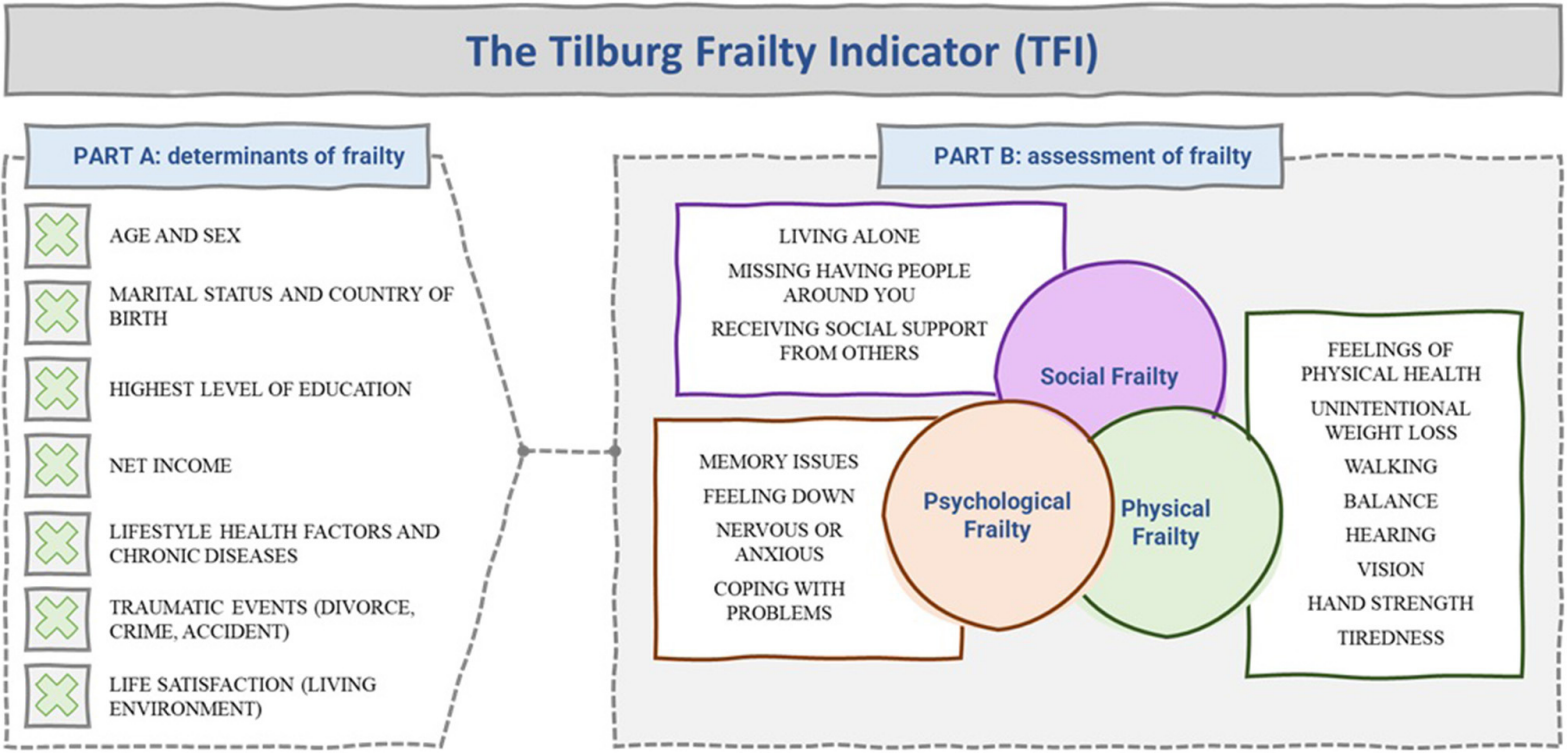
Tilburg Frailty Indicator (TFI) (18–20).

Another noteworthy frailty measure is the Tilburg Frailty Indicator (TFI) [see (18–20)]. The TFI takes the foundational elements of the cumulative model of frailty and extends the construct to explicitly measure psychological and social elements. However, the TFI distinguishes itself from other cumulative model measures not only because of its focus on psychological and social elements of frailty, but also because it does not contain questions referring to disability nor disease. The typical questions asked in the user-friendly and self-reported TFI are summarized in Figure 4. The TFI also has the important benefit of attempting to measure the determinants of frailty, not only assessing if someone is frail.

The frailty construct shows promise as a relatively quick, affordable and effective measure of the early signs of geriatric syndromes and premature aging. The broad application of such a measure in PEH could offer improved detection of premature geriatric conditions and early support to a group for whom health engagement can be a challenge. However, there are a vast range of different frailty measures and, as such, there is no gold standard assessment approach. This increases the complexity of applying and interpreting frailty measures. Further, much of the debate about the value of the frailty construct has not considered the application of the concept in the context of PEH; a group at risk of accelerated aging and the premature onset of geriatric conditions, with significant barriers addressing these conditions. There is ultimately a lack of research on the use of the frailty construct to assess and support PEH. Given the potential value of the frailty construct to predict adverse outcomes, its relative ease of use and potential capacity to measure the upstream determinants of geriatric conditions, including social and psychosocial factors, a synthesis of the frailty construct in the context of PEH is greatly needed to query the value of the construct for this group. This is particularly important as the number of older PEH grows rapidly across the world and, without intervention, will continue to do so over the coming decades.

### Objectives

The aim of this study is to conduct a rapid review on the application of frailty in adult PEH. Specifically, this review aims to synthesize the findings of studies that have measured frailty or related geriatric constructs and investigated factors that contribute to frailty in PEH; which may in turn highlight existing opportunities for early intervention.

This rapid review aims to answer the following questions:

1. Do PEH experience higher levels and/or earlier onset of physical frailty and other frailty-related geriatric conditions when compared with ‘housed' populations?
2. What are the most significant cognitive, psychological, and social determinants of frailty and other frailty-related geriatric conditions in PEH?

## Methods

We conducted a rapid review which provides a streamlined version of a more traditional systematic review (21). Rapid reviews attempt to accelerate the review process, resulting in timely outputs that act as a rigorous summary of the literature rather than an in-depth synthesis (22). The adaptive methodology supported in rapid reviews suited the aims of this research, i.e., investigating the emerging and dynamic nature of the frailty construct [see (8)].

For the purposes of this work, methods included: independent and systematic searches by two researchers (RM and SP). Both screeners were independently involved in applying inclusion/exclusion criteria, underpinned by a comprehensive review strategy, for all search results using Covidence software. Where there was disagreement between the two screeners, the senior author (AW) screened these results. AW also acted as a triple screener of the titles and abstracts for 10% of studies to ensure fidelity of the process. Screening was followed by a thorough data extraction process audited by all authors to ensure consensus.

### Search strategy

A search strategy was developed based on three intersecting concepts: Aging, homelessness and frailty. Given our interest in (a) accelerated aging and (b) cumulative geriatric difficulties, we also incorporated search terms which would capture these concepts, namely: premature, accelerated, onset and geriatric.

#### Data sources

Three electronic databases were searched: Medline, Embase and PsycINFO.

#### Original search query

(Old^*^ OR elder^*^ OR geriatric^*^ OR gerontol^*^ OR aging OR aged) AND (homeless^*^ OR PEH OR unhoused) AND (health^*^ OR frail^*^ OR disease^*^ OR infection^*^ OR treat^*^ OR illness^*^ OR decline OR dementia OR functional OR onset OR premature OR accelerated).

#### Review criteria

We reviewed primary research papers studying PEH and which assessed frailty or frailty-related concepts between 2000 and 2021. Frailty-related concepts included studies on geriatric syndromes in PEH as well as studies which explicitly looked at an accumulation of deficits across two or more psychological, social and physical domains, which could have been reasonably included into a cumulative model of frailty. The latter search strategy required a level of interpretability by the research team. To ensure quality control and consistency the researchers implemented a further rule that to include a paper, it must:

- Explicitly involve a frailty measure or framework, *OR;*
- Measure cumulative geriatric syndromes or outcomes with high conceptual overlap with frailty (e.g., functional dependence, falls, incontinence), *OR;*
- Measure at least one physical geriatric deficit or condition *AND* at least one measure of either psychological, cognitive *OR* social burden.

It was deemed important to include the final point given the under-recognized contribution of social and psychological disadvantage in premature aging and physical frailty (23–25), and because the capacity to measure social and psychological deficits may enable early intervention or prevention of frailty (6).

For the purposes of this study, we defined homelessness to include primary, secondary and tertiary forms of homelessness. This excluded people in marginal housing, including permanent supportive housing. An exception was made when studies incorporated samples with both homeless and precariously housed individuals, in which case a study was included.

This study aimed to investigate the onset of frailty in adult PEH and as such we did not actively define a minimum age threshold for presenting with geriatric conditions apart from the requirement that study sample populations were aged 18 or over.

#### Data extraction

Summary study information was extracted into a data workbook after a full text review. Data columns included Author(s); Year; Title; Journal; Location; Study design; Design Comments; Target population and/or setting; Sample Size; Age (Mean); Female (%); Frailty tool(s); Frailty tool(s) comments; Other tool(s) used; Study Aims; Main implications and/or insights. A summary version of the data extraction can be found in Table 1.

**Table 1:** Study characteristics.

**References**	**Year**	**Location**	**Study design**	**Target population(s)**	**Sample size**	**Age (X)**	**Female (%)**
Brown et al. (26)	2013	Boston, USA	Cross-sectional study	≥50 years PEH from emergency, transitional, and day shelters	250	56	19.20%
Brown et al. (27)	2012	Boston, USA	Cross-sectional study	PEH adults aged 50–69 recruited from emergency, transitional and day shelters	247	56	19.80%
Brown et al. (28)	2017	Oakland, USA	Cross-sectional study	≥50 years PEH from shelters open to older adults, all free and low-cost meal programs, recycling centers, and areas where adults slept unsheltered	350	58^*^	22.90%
Gicas et al. (29)	2020	Vancouver, Canada	Prospective cohort study	≥18 years PEH or precariously housed	375	44^*^	22.00%
Gicas et al. (30)	2021	Toronto, Canada	Prospective cohort study	≥18 years PEH, meeting criteria for a mental disorder (with or without a substance use disorder)	349	40	31.50%
Jutkowitz et al. (31)	2019	USA	Cross-sectional study	Veterans in a nursing home with a record of homelessness in the year prior to their nursing home admission	3,355	63	4.60%
Kiernan et al. (32)	2021	Dublin, Ireland	Cross-sectional study	PEH in an acute hospital inpatient facility ≥ 18	65	47	32.30%
Mahmood et al. (33)	2021	San Diego, USA	Cross-sectional study	PEH between 18 and 89	100	49	19.00%
Moquilazza-Risco et al. (34)	2015	Lima, Peru	Cross-sectional study	PEH ≥ 60 years	302	72	17.00%
Patanwala et al. (35)	2018	Oakland, USA	Prospective Cohort Study	PEH ≥ 50 at a community-based agency serving low-income older adults, overnight homeless shelters, low-cost, a recycling center, and places where unsheltered homeless adults stayed	350	59^*^	19.80%
Rogans-Watson et al. (36)	2020	London, UK	Cross-sectional study	Hostel for single PEH ≥30 years with complex needs	33	56	9.00%
Rogoz et al. (5)	2016	Sydney, Australia	Cross-sectional study	PEH ≥45 recruited from shelters (32.8%); hospital (12.9%); hostel (53.2%); and housing agencies (1.1%)	171	55	16.00%
Salem et al. (25)	2013	Los Angeles, USA	Cross-sectional study	PEH ≥40 without acute psychotic hallucinations and psychosis	150	52.4	50.00%
Salem et al. (23)	2019	Los Angeles and Pomona, USA	Cross-sectional study	Homeless ex-offending women; 18–65 with past drug use from community-based sites	130	39	100.00%

^*^Median age.

## Results

**Figure 5:**
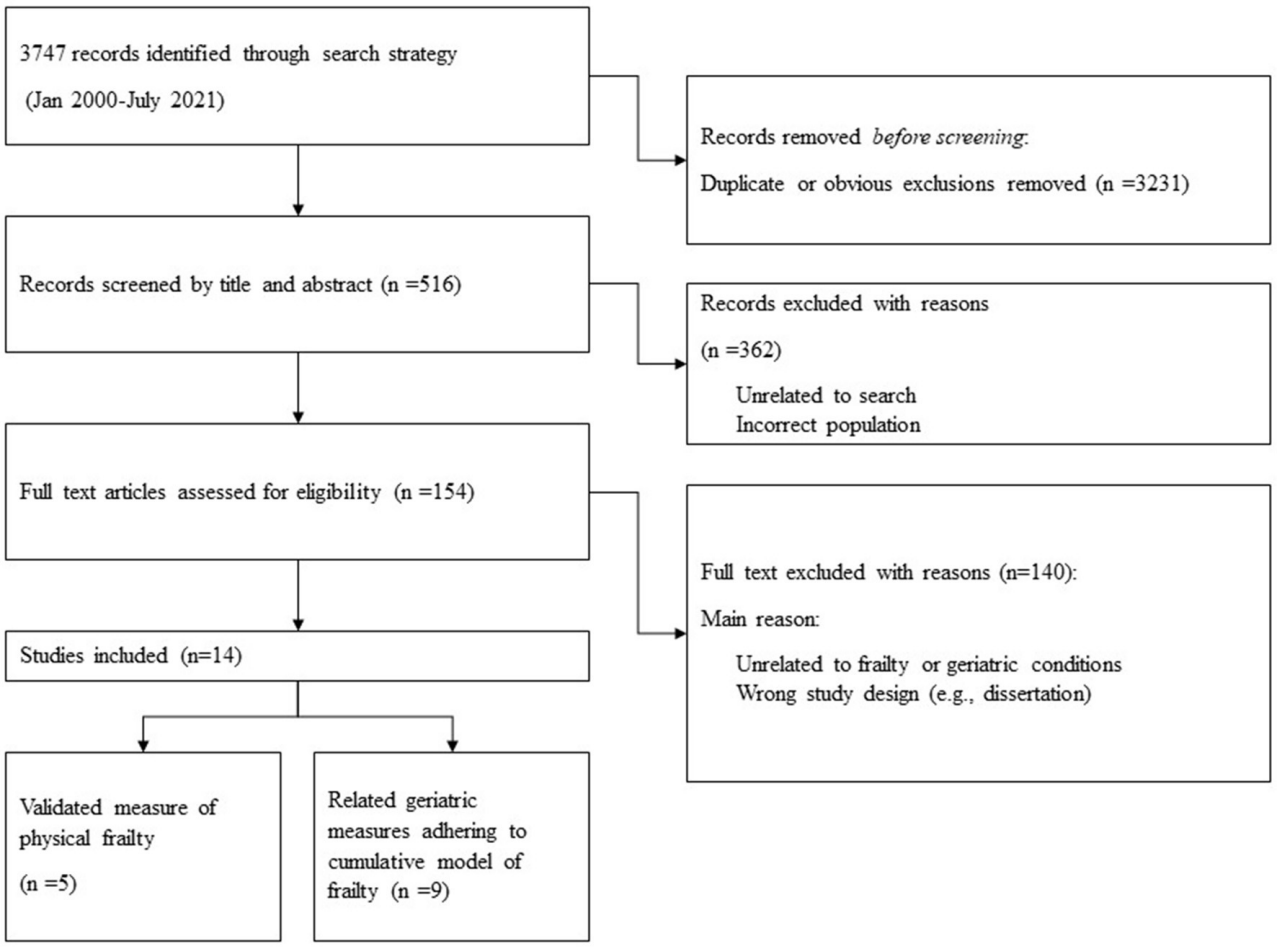
PRISMA diagram.

Our initial database search yielded *n* = 3,747 papers. After removing duplicates and obvious exclusions, *n* = 516 papers were included for abstract screening and a further *n* = 154 were included for full screen review. Through our final search strategy and extraction process we identified *n* = 14 research papers that met the study criteria. Of these papers *n* = 5 used validated measures of physical frailty, and the other *n* = 9 adhered to cumulative model constructs of frailty (*defined above*) (Figure 5). All papers were cross-sectional or cohort studies. All papers were from anglophone countries with the exception of one paper from Peru (34). There were a diverse range of average ages across the studies—from 39 to 72 years. There were also some noticeable gender differences across the study samples; only two of the studies had more than 33% female participation. However, one of these papers (Salem et al., 2019) included only female participants. Finally, although there was some variance in the definition of PEH, all papers sampled participants from cohorts that conformed to our broad definition of homelessness.

### Prevalence of physical frailty and other frailty-related geriatric conditions among PEH

The prevalence of physical frailty was measured directly in five studies of PEH (23, 25, 26, 32, 36). A further three studies (27, 28, 34) directly reported on geriatric conditions that were related to physical frailty. Although these papers did not explicitly measure frailty, the findings from these papers either directly or indirectly conform to a cumulative deficit model of frailty and thus highlight important geriatric difficulties for PEH. Findings are summarized in Table 2.

**Table 2:** The prevalence of frailty in PEH.

**References**	**Sample size**	**Age (X)**	**Frailty (%)**	**Frailty tool**	**Other key findings**
Rogan's-Watson et al. (36)	33	56	55% (i.e., 2.6/5)	Fried's phenotype	Frailty was also measured in the study using the Edmonton frail scale (55%) and Clinical Frailty Scale (48%)
Kiernan et al. (32)	65	47	23.3%	Clinical Frailty Scale (CFS)	Only one participant obtained a score of one (very fit) and only 31.7% were classified as being robust or “non-frail.” The distribution of frailty scores was higher in females than males (*p* = 0.023) and there was no difference in frailty scores between age groups (*p* > 0.05)
Brown et al. (26)	250	56	16%	Fried's phenotype	Over 70% of participants reported having two or more geriatric conditions. Only 8.4% of the sample reported having no geriatric conditions and more than half reported they had fallen in the past year (53.4%). Nearly half had sensory impairment defined as hearing and/or vision impairment, and nearly half also reported urinary incontinence
Brown et al. (27)	247	56	N/A	Cumulation of geriatric syndromes	After multivariate adjustment, syndromes including functional and mobility impairment, depression, visual impairment and urinary incontinence, all indicative of cumulative frailty, were statistically more likely in PEH compared to matched samples (further discussed in next section)
Brown et al. (28)	350	58^*^	N/A	ADLs and IADLs	Over a third of all participants (38.9%) reported difficulty performing one or more ADLs and nearly one-fifth (17.1%) had difficulty performing three or more ADLs. Nearly half (49.4%) of the sample reported difficulty performing one or more instrumental activities of daily living (IADLs)
Moquilazza-Risco et al. (34)	302	72	N/A	KATZs	Nearly half the sample (48.9%) were at least partially dependent. Functional dependence was measured using the KATZ's index of independence, similar to a traditional ADL measure. In addition, during a logistical regression analysis, it was found that women were more likely than men to become functionally dependent
Salem et al. (23)	130	39	Physical psychological social	Tilburg Frailty Indicator (TFI)	37% had one frailty domain with a score above the median. Twenty-one percent had two frailty domains with domain scores above the median and 7% had all three domains with scores above the median. The number of domains with scores above the respective median was not significantly related to age
Salem et al. (25)	150	52.4	54%	Frailty Index (FI)	When comparing FI frailty scores to the holistic frailty framework among vulnerable populations (FFVP) measures (discussed further in Psychosocial section), there were significant moderate negative correlations between frailty and resilience, social support and nutrition

^*^Median age.

### Physical frailty in PEH in the context of broader population studies

Of the eight papers which reported the rates of physical frailty and other frailty-related geriatric conditions in PEH, four were indirectly compared to frailty rates in other cohorts. In Rogans-Watson et al. (36), as assessment criteria were based on methods used in the English Longitudinal Study of Aging (ELSA), comparison to population data was feasible (37). When compared to ELSA data, the average frailty rates (2.6/5) of the PEH sample (average age 56) were equivalent to the mean for an 89-year-old in the general population in England (36).

In another study by Brown et al. (27), geriatric syndromes were measured using the same sample of older PEH as Brown et al. (26). Findings were subsequently compared with population-based cohorts to investigate differences in the prevalence of geriatric issues. When matched with Maintenance of Balance, Independent Living, Intellect, and Zest in the Elderly (MOBILIZE) of Boston Study (MBS), PEH were less likely to report good, very good or excellent health (*p* < 0.001). Rates of physical frailty were significantly higher for PEH than the MBS cohort (16% vs. 10%) (*p* < 0.001). In Brown et al. (28), the authors compared their findings with the MBS cohort (27, 38, 39), as they did with a different PEH sample in 2012 (27). When compared to the MBS study sample (*n* = 765, mean age of 78.1), rates of several geriatric conditions were higher in the much younger PEH sample (median age 58). A second comparison was made with a cohort of community-dwelling adults aged 65 and older (mean age 71.7 years) with a very low-income (40). Low income was defined as income < 200% of the United States poverty level. When compared to this much older and low-income group, PEH still had a significantly higher prevalence of falls (33.7% older PEH vs. 21.9% older adults living in poverty), visual impairment (45.1% vs. 12.0%), urinary incontinence (48.0% vs. 29.5%), and depression (38.3% vs. 11.3%) (28).

Finally, in Moquillaza-Risco et al. (34), the authors compared their findings with the Health, Welfare and Aging Survey (SABE, Spanish acronym), which was conducted in several Latin American and Caribbean countries (41). The SABE study indicated that between 10% and 25% of older survey participants had at least some kind of difficulty with ADLs and IADLs (41). This was noticeably lower than the 50% prevalence of *at least* partial functional impairments found in Moquillaza-Risco et al. (34).

### Cognitive impairment and functional issues in PEH

An important finding highlighted by four studies (5, 29, 33, 34) in this rapid review, summarized in Table 3, is that many PEH present with significant cognitive deficits at relatively young ages.

**Table 3:** Summarizing the relationship between cognitive impairment and functional dependence in PEH.

**References**	**Cognitive tool**	**Cognitive impairment**	**Relevance to frailty and adjacent age-related decline**
Rogoz and Burke (5)	Mini-mental state examination (MMSE)	49.1% scored 26 or less, indicating evidence of cognitive impairment	Of PEH who scored as cognitively impaired, nearly 80% self-reported having mental health problems; and likewise mental health problems greatly increased the odds of also having cognitive impairment [OR = 7.16, 95% CI = (2.31, 22.19)]
Moquillaza-Risco et al. (34)	Pfeiffer's test	Mild cognitive impairment = 30.7%. Moderate cognitive impairment = 23.2%. Severe cognitive impairment = 12.5%	In a logistical regression model the probability of partial functional dependence, measured by the KATZ index, increased greatly with the severity of cognitive impairment, highlighting the interrelationship between functional impairment and degree of cognitive impairment
Gicas et al. (29)	Hopkins verbal learning test-revised Stroop test for inhibitory control	At baseline evaluation: 68.1% scored at or below the cut-off for verbal learning 62.9% scored at or below the cut-off for verbal memory. 10% scored as clinically impaired for inhibitory control	Survival analyses established that better inhibitory control was associated with a 6.6% decreased risk of mortality in the sample, and this protective effect of cognition became larger by 0.3% for every additional year of life, controlling for co-occurring chronic medical illnesses
Mahmood et al. (33)	Montreal cognitive assessment (MoCA)	65% impairment rate with a standard cut-off score of 26 and 30% with a cut-off of 23	Nearly half of the participants (47%) met criteria for functional impairment and 17% of the sample were not expected to be capable of living independently. Participants' functional abilities were assessed using the University of California, San Diego, Performance- Based Skills Assessment–Brief (UPSA-B) which measures functional capacity by asking participants to role play everyday tasks

In an Australian cross-sectional study by Rogoz and Burke (5) nearly half the sample indicated evidence of cognitive impairment. Further, in Moquillaza-Risco et al. (34), only 33.6% of the sample were assessed as having normal cognitive function. The likelihood of functional dependence increased with age for all degrees of cognitive impairment, except for severe cognitive impairment, where the likelihood of dependence was close to 100% (i.e., fully dependent) at all ages.

In addition, the prevalence of cognitive impairment was fourfold higher among older PEH than among the SABE sample (34). These findings were reinforced in Gicas et al. (29), a nine-year community based longitudinal study of homeless and precariously housed people with a median age of 44 (age range 23–68). The study investigated the relationship between cognitive health and mortality. Across the study period, subsequent decline in verbal memory was most notable for individuals with a history of traumatic brain injury or alcohol dependence at baseline. Significant decline in inhibitory control was observed in the study, with greater decline for those who died during follow-up and for those who spent more years living in an impoverished environment. In the final model adjusted for comorbidities, inhibitory control remained a significant predictor of mortality.

In Mahmood et al. (33), there were significantly lower cognitive function scores (i.e., higher impairment rates) than expected in the general population (*p* = 0.001). MoCA scores were significantly associated with UPSA-B scores (*p* < 0.001), highlighting the strong connection between cognitive and functional performance, and reinforcing the interrelationship between the two (33).

### The potential relationship between other psychosocial factors and frailty

The impact of a range of different psychosocial factors in PEH, and how they contribute to frailty, functional dependence and other geriatric conditions was reported in eight studies (23, 25, 26, 28–31, 35).

High levels of drug and alcohol dependence among PEH was found in numerous studies. In Brown et al. (26), drug use was associated with a 2.3 times higher total number of geriatric syndromes. In Brown et al. (28), nearly three-quarters (71.3%) of partipants had a history of mental health problems and more than half had a lifetime alcohol and/or drug use problem. In Gicas et al. (29), alcohol dependence was associated with greater impairment in learning, memory and motor functions. It was considered an important factor in the accelerated cognitive aging of this cohort (29). Similar patterns were observed in a cross-sectional study of *n* = 3,355 American veterans who were homeless in the year prior to their community nursing home admission (31). At the time of nursing home admission, participants were more likely to have had a diagnosis for a substance use disorder [Adjusted Relative Risk (ARR) = 2.18; 95% CI = (2.05–2.31)], dementia (ARR = 1.14; 95% CI = 1.04–1.25) and a mental health condition [ARR = 1.49; 95% CI = (1.45–1.54)] compared to those who were stably housed (31).

Further research has highlighted the relationship between a range of novel environmental and psychosocial factors and physical functioning in PEH. An important psychosocial finding in Gicas et al. (29) was that longer time living within an impoverished neighborhood was associated with greater decline in inhibitory control. The authors concluded that this finding may reflect “*the cumulative effects of socioeconomic disadvantage, unsafe living conditions and social stressors. Lack of community resources for cognitive enrichment in day-to-day life may also contribute” [(**29**), p. 6]*.

A study by Patanwala et al. (35) of PEH aged 50 and over (median age of 59 years) found over half (57.6%) of the participants had psychological symptoms and 26.5% had ‘high regret'. In a multivariate regression model, it was established that being a woman [Adjusted OR = 2.54, 95% CI = (1.28–5.03)], having a history of childhood abuse [AOR = 1.88, CI= (1.00–3.50)], cannabis use [AOR = 2.59, CI = (1.38–4.89)], multimorbidity [AOR = 2.50, CI = (1.36–4.58)], anxiety [AOR = 4.30, CI = (2.24–8.26)], hallucinations [AOR = 3.77, CI = (1.36–10.43)], and loneliness [AOR = 2.32, CI = (1.26–4.28)] were all associated with moderate to high physical symptom burden. The authors also found an overall prevalence of loneliness (39.6%) higher than the estimated prevalence among older adults in the general population [estimated community prevalence reported from Ong et al. (42)]. The authors concluded that the high prevalence of loneliness in aging PEH could be an important contributor to functional decline in this group.

In a Canadian sample of 349 homeless adults with serious mental illness, and a relatively young average age of 39.8, the relationship between community functioning, cognitive health, Quality of Life (QoL), resilience and experiencing homelessness were investigated (30). After adjusting for select risk and protective factors, composite indices of verbal learning and memory, processing speed and cognitive flexibility, were all positively associated with community functioning, but not with QoL, over a 6-year period study period. Greater individual resilience levels were independently associated with better QoL. Cognition was the predominant predictor of community functioning, whereas select risk and protective factors (childhood adversity and resilience, respectively) were specifically associated with QoL.

The frailty framework among vulnerable populations (FFVP) is a latent construct proposed by Salem et al. (25) which incorporates social and psychological elements into a holistic framework of frailty designed specifically for assessing and understanding marginalized populations.

The FFVP was tested or applied in two studies in this review (23, 25). In Salem et al. (25) a group of older PEH (average age 52.4) were assessed across a number of situational, health-related, behavioral, resource, biological, and environmental factors; designed to capture physical, psycholgical and social frailty. These assessments were subseuqently compared to a traditional frailty measure [Rockwood's Frailty Index (FI)], where the prevalence of frailty was 54%. When comparing FI frailty scores to the holsitic FFVP measures through a Pearson (r) bivariate correlation, significant moderate negative correlations between frailty and resilience (*r* = −0.395, *p* < 0.01), social support (*r* = −0.377, *p* < 0.01), and nutrition (*r* = −0.652, *p* < 0.01) were found. In the final model, age, gender, health care utilization, nutrition, and resilience were significantly related to frailty. The squared multiple correlation coefficients was 0.542, suggesting that 54.2% of the variance in frailty can be predicted by and age, gender, health care utilization, nutrition, and resilience (25).

In another study by Salem et al. (23), a sample of relatively young, formerly incarcerated women experiencing homelessness (average age 39 years), were assessed for physical frailty, psychological frailty and social frailty. These frailty outcomes were measured using the Tilburg Frailty Indicator (TFI) [see (18–20)]. In the sample, those who had a greater number of prior violent offenses had higher levels of physical frailty (*p* = 0.001); participants with a higher PTSD symptom score (*p* = 0.012), or a lower tangible support score (*p* = 0.001), had higher levels of physical frailty. Greater bodily pain was also associated with greater levels of psychological frailty (*p* = 0.036). Those with a higher drug dependency score had higher physical and psychological frailty (*p* = 0.047 and *p* = 0.033, respectively) and those who used a greater number of drugs had a higher likelihood of being socially frail (*p* = 0.009). Higher emotional regulation difficulty scores were also associated with higher levels of social frailty (*p* < 0.001) (23).

## Discussion

The aim of this rapid review was to examine frailty in adult PEH. The findings establish collective evidence that frailty, either defined as phenotypical frailty, multidimensional frailty (*i.e., the TFI*) or the accumulation of relevant geriatric conditions, signs and symptoms (i.e., *indexed frailty/frailty scales*), presents earlier and at higher rates in PEH than community-dwelling cohorts. In some studies, the comparisons are quite stark. PEH aged in their 40s and 50s had similar frailty scores and geriatric conditions as people aged in their 70s and 80s (26, 27, 32, 36). These differences remained when PEH were compared to a cohort with very low incomes (28, 40). This high burden of early-onset geriatric difficulties provides further evidence that PEH are at risk of accelerated aging (7) and consequently premature functional decline, disability and death.

This review also synthesized novel insights regarding the antecedents of frailty in PEH, namely that psychosocial and structural determinants of health and wellbeing are associated with frailty onset and severity. For instance, loneliness (35), living in an impoverished neighborhood (29), resilience (25, 30), being female (32) and drug and alcohol use (23, 26, 31) were all associated with functional dependence and decline in PEH. However, given most papers in this review were cross-sectional studies, it is not possible to make any general claims regarding the causal relationship between upstream determinants and frailty. This points to the urgent need for more cohort studies in this area. Regardless, these findings build upon previous work on early morbidity, mortality and accelerated aging in PEH (1) by mapping health decline to a validated construct, frailty; thereby providing a richer analysis of unequal aging and aging-related decline in PEH (8).

A notable difficulty for many aging PEH is cognitive impairment, which is associated with a range of negative outcomes, including early functional dependence, reduction in autonomy and reduced mobility. Rates of global cognitive impairment in PEH ranged from 25% to 65% across the studies in this review. Gicas et al. (29) found cognitive deficits, specifically in executive functioning, to be particularly debilitating for aging PEH. These deficits appeared for PEH in their 40s, decades earlier than healthy community-dwelling participants (29, 43). However, when interpreting these results it is important to note that high impairments scores in PEH could be related to the high incidence of mental illness such as depression or other psychiatric disorders in many of the PEH cohorts tested. For example, in a sample of PEH with cognitive impairments, 88.8% self-reported mental health problems (5). This high prevalence of mental health issues can have effects on cognitive performance scores and potentially overstate cognitive deficits. In addition, other upstream factors such as cultural or educational factors (including low literacy) are known to mediate cognitive performance scores in marginalized groups. These confounders need to be addressed in future research. Regardless, cognitive impairments in PEH appear to have an important, and interconnected, relationship with functional decline and dependence (33, 34), and these issues can emerge concerningly early in life.

This review found that the combination of poor mental and cognitive health difficulty greatly increases the risk of comorbid functional decline (5, 33, 34). These findings are reinforced by a large cross-sectional study (*n* = 1,500) of PEH with an average age of 41.1 (44). Stergiopoulos et al. (44) established that PEH with mental illness experience significant neurocognitive impairment; with nearly three quarters of PEH with mental illness showing evidence of neurocognitive impairment (44). Collectively, these findings indicate that cognitive impairment (both with or without mental health commodity) is an important contributor to functional decline in aging PEH, and subsequently the accelerated aging and premature frailty of the group. Efforts to assess cognitive health in PEH should be prioritized and seen as a vital underpinning to broader health and social care efforts to support aging PEH. Given the premature aging of the group, cognitive assessment efforts should be considered for PEH in their 40s and 50s. Further, given the relationship between functional dependence, cognitive impairment and other mental health issues, cognitive assessment should be carefully considered in the broader context of a person's physical and mental health and the high risk of comorbidities (including confounding disorders) across these domains.

Another recurrent theme in this review is the impact that drug and alcohol use and dependence can have on the health of PEH (26, 29, 30, 36). Drug and alcohol use can cause decline in cognitive functioning in PEH (29), particularly executive functioning. Chronic drug and alcohol use can also increase the risk of developing frailty by negatively impacting nutrition (45, 46) and sleep quality (47, 48). Further, drug and alcohol use by somebody once they are frail also increases the risk of serious falls (49), incontinence (27) and hospitalization (50). Prioritizing drug and alcohol assessment, treatment and management as a preventative measure to reduce the risks of accelerated aging and frailty in later life for PEH is of key importance.

Patanwala et al. (35) established that in an aging sample of PEH, loneliness was an independent predictor of both functional decline and mortality; and loneliness rates were higher in PEH than older community dwelling adults. Loneliness is being increasingly recognized as an important determinant of health and wellbeing. It is a key predictor of depression, substance disorders and cognitive decline in older people (51) and feelings of loneliness are of particular concern for those who are at increased risk of social disconnectedness and deprivation of genuine connection with family, friends and communities (52). Such risks are likely heightened for many aging PEH who live alone or in unpredictable environments. For instance, only 9.6% of the PEH sample in Moquillaza-Risco et al. (34) reported having a close relative. Loneliness appears to be an important consideration in the accelerated aging of PEH and warrants further attention (35). Importantly, with the exception of more holistic measures of frailty such as the TFI, the majority of traditional frailty measures do not adequately capture measures of social frailty like social exclusion, loneliness or sufficient social supports.

Although frailty measures tend to focus mainly on physical health deficits, this review has highlighted the importance of psychological, cognitive, psychosocial and environmental factors in relation to both the determinants of frailty, and the severity of frailty itself. For instance, using the frailty framework among vulnerable populations (FFVP) it was established that educational attainment, nutrition, greater number of years homeless, being divorced, poorer emotional regulation and those who identified as either being Black or female all were significantly associated with social, psychological and/or physical measures of frailty (23, 25). These findings are important as little research has been conducted into the etiologies of accelerated aging or premature frailty in PEH, and even less on intersectional aspects for this group. These findings also reinforce the upstream and structural social factors that often contribute to the cumulative health and social difficulties experienced by PEH. In this regard, frailty frameworks such as the FFVP and frailty measures such as the TFI- which actively include measures of psychological, cognitive and social frailty—appear relevant for PEH. However, there remains no gold standard assessment of frailty, and many measures (including the TFI) remain mostly underutilized and unvalidated for PEH and other marginalized groups at risk of accelerated aging. For instance, as summarized in Table 2, in the five studies that directly measured frailty captured in this review four different frailty tools were used, with only two of these directly collecting data on psychological or social issues. It appears important to further explore the psychosocial and environmental contributions of frailty within marginalized groups in the context of the broader literature on physical frailty to ensure research consistency and clinical usefulness.

### Limitations

This review has some limitations. None of the papers in this review examined the interrelationship between pathophysiological dysfunction at a biological level and the environmental or lifestyle determinants that may cause cellular deterioration. However, by measuring frailty and other related geriatric conditions and their associations with social, psychological and cognitive difficulties, a number of studies examined the contribution(s) of certain factors or determinants which appear to modify (accelerate) the aging process, i.e., functional decline, early mortality, etc.

As seems to be the case with much of the literature on frailty, which specific factors are most important in a single study depends on the way frailty is defined, measured and applied; and how these factors relate with the biological processes of aging, and in what context, is not always clear. This is certainly a barrier to the application of frailty research, but not necessarily a fatal one. As shown through this rapid review, you can analyze differential applications of the construct concurrently and identify patterns and overlap. An example of this is the lenient search strategy applied in this review to capture cognitive and psychosocial difficulties which have theoretical and practical links to frailty yet would not usually be included in traditional frailty research. Regardless, the fundamental differences between the two dominant approaches to frailty, as well as contemporary multidimensional measures and framework, have caused considerable practical and theoretical barriers to applying the construct over the last two decades, including disparate measures of predictive validity, different minimum data requirements and variable administration methods (53). Besides a lenient and dynamic search strategy and definition of frailty, no attempt to reconcile these differences was made in this review. Moving forward, a standardization of concepts should be attempted. This is increasingly important given the emergence of measures like the TFI and conceptual frameworks like the FFVP; which although add important contributions to the psychosocial and environmental elements of frailty, also increase the confusion surrounding the original construct.

Finally, the distinction between the concept of frailty and other related constructs, namely multimorbidity, is often difficult to define. The major distinction in the current literature is that multimorbidity refers exclusively to the coexistence of clinically manifest diseases, whereas frailty refers to an increased vulnerability to stressors which could include symptoms, signs, diseases, disabilities or laboratory, radiographic or electrocardiographic abnormalities (54). Although there is some attempt by the authors of this paper to distinguish between frailty and multimorbidity through a clear and comprehensive search criterion, the overlap between these concepts is substantial and requires further attention.

## Implications

This rapid review has important implications for service provision. Service providers and clinicians should be aware that PEH aged in their 40s and 50s, or even earlier [e.g., (32)] can be physically frail and experience geriatric conditions as well as cognitive and functional impairments. For PEH, earlier onset geriatric conditions and concurrent chronic diseases, mental health issues and psychosocial problems are often accompanied by poor access to appropriate and effective treatment (5). This contributes to recurrent emergency department presentations (55) and high hospital readmission rates for PEH (56); with nearly four times the odds of being readmitted within 30-days as compared to low-income matched control participants (57). These difficulties further increase the complexity and cost of health treatment (50) and reduce the likelihood of health improvement, which underscores the importance of early intervention for PEH.

Importantly, the findings and recommendations presented in this rapid review should be seen as complementary to, and not a substitute for, long term housing strategies to reduce homelessness. Interventions to ensure stable and safe housing are essential supports for aging PEH to access community and/or aged care services, as well as reduce the cumulative health and social disadvantages that people who are currently homeless experience. As such, a suitable approach would be to strive for housing for PEH in parallel with more holistic, and equitable, service offerings to support PEH health and wellbeing.

To assist with timely detection of health issues, which may facilitate early intervention or even prevention of frailty and geriatric conditions before they emerge or progress (7), a presentation for one condition should trigger comprehensive health screening, including social determinants of health. Given the rates of frailty at a relatively young age for PEH, screening should be initiated early and often in this population. As highlighted by this review, a focus on co-occurring psychosocial and cognitive factors would be beneficial. Important psychosocial contributors to frailty and/or functional decline in PEH include cognitive decline (5, 29, 33, 34), drug and alcohol use (26, 29, 36, 58) and social isolation and loneliness (35). These factors are particularly important to detect for aging PEH as they can potentially lead to early modification and/or rehabilitation which may support proactive intervention in frailty pathways. It is recommended that research and practice exploring frailty in PEH incorporate minimum data on these three factors and explore interventions in these spaces. Measures such as the TFI are promising in this regard, however, require further research to establish psychometric validity for PEH at risk of accelerated aging. Regardless of what frailty tool is used, it should be considered as part of a broader suite of supports to reduce and manage frailty which often include exercise and nutrition interventions, sensible housing strategies and traditional geriatric services.

Finally, this review reported the structural, upstream and often intersectional determinants which can contribute to frailty, such as living in an impoverished neighborhood, educational attainment, being Black or female. It is important to appreciate that many of the contributors to accelerated frailty in PEH, including functional and cognitive decline, drug and alcohol use and loneliness are often steeped in longer-term social difficulties and likely require more holistic and/or multidimensional intervention strategies (such as housing). Acknowledging these factors, and better understanding the dynamic and multidimensional burden facing PEH, which can manifest as accelerated aging and frailty conditions, is an important first step to better supporting the health and wellbeing of PEH.

## Author contributions

SP, PC, AW, and KR contributed to the original conception and design of the study. SP, RM, and AW screened studies. RM and SP conducted the analysis, with guidance from AW, PC, and KR. RM wrote the first draft of the manuscript and final manuscript. YH wrote sections of the manuscript and edited the first draft. All authors contributed to manuscript revision, read, and approved the submitted version.
